# Natural Bioactive Compounds in Cosmeceuticals: Challenges and Future Perspectives

**DOI:** 10.3390/ijms27219709

**Published:** 2026-10-29

**Authors:** Firoozeh Kavosh Tehrani, Marco Masi

**Affiliations:** Department of Chemical Sciences, University of Naples Federico II, Complesso Universitario Monte S. Angelo, Via Cintia, 80126 Naples, Italy; firooozeh.kavoshtehrani@unina.it

**Keywords:** plant metabolites, cosmetic application, natural bioactive compounds, natural cosmetics

## Abstract

The global cosmetic market is experiencing dramatic growth, and it is shifting significantly toward the use of natural substances rather than synthetic ingredients in cosmetic formulations. Natural bioactive compounds, obtained from plants, are used in the modern cosmetic industry in order to enhance skin appearance and provide biological bene-fits. These compounds include flavonoids, terpenoids, steroids, fatty acids, and vitamins with anti-aging, antioxidant, anti-inflammatory, and antimicrobial properties. Most previous studies have focused on plant extracts and their general effects on human skin, without bridging the gap between systematic classification of bioactive compounds and their cosmetic applications. Therefore, the aim of this article is to provide a novel framework for bioactive compounds which are utilized in cosmeceuticals. Additionally, it discusses biological activities of each compound associated with skin well-being, its applications in the cosmetic industry, as well as its general challenges and limitations in cosmetic formulations.

## 1. Introduction

Human skin is the largest organ of the body and the first line of defense against environmental stressors such as UV radiation, pollution, microorganisms, and chemical irritants. Regular exposure of the skin to these factors leads to oxidative stress, inflammation, extracellular matrix degradation, and skin aging. Therefore, there is a growing need for effective products that can maintain skin homeostasis. Moreover, disruption of the skin barrier increases transepidermal water loss (TEWL), increases inflammation, and reduces skin hydration. Therefore, the importance of effective compounds in cosmetic products that help maintain the integrity of the skin barrier cannot be overemphasized [1].

The global cosmetics industry is considered one of the fastest-growing industries in the world in recent years [2]. There has been a significant rise in the number of consumers who are interested in healthy skin, keeping a youthful appearance, and using safe and natural products. Therefore, novel cosmetic products are being developed to meet these demands. These products are designed not only to improve skin appearance but also to protect the skin from environmental damage, keep the skin healthy, and even help improve some skin disorders [1]. This trend led to the introduction of the term cosmeceuticals, which bridges the gap between conventional cosmetic products and pharmaceuticals [3,4]. In addition, some synthetic cosmetic ingredients may cause side effects, and as a result, consumers increasingly prefer products containing natural ingredients. While many synthetic cosmetic formulations are safe when used in controlled amounts, the use of natural compounds, especially those derived from plants, has increased in the beauty industry [4]. Therefore, new cosmetic ingredients contain different types of natural bioactive compounds derived from plants to improve skin appearance and regulate its physiological processes.

Natural bioactive compounds are widely applied in cosmetic formulations owing to their antioxidant, anti-inflammatory, antimicrobial, skin-brightening, anti-aging, moisturizing, UV-protective, and collagen synthesis-stimulating properties, as well as for skin barrier repair [3]. Additionally, they can help to decelerate the skin aging process and protect it from environmental damage by scavenging free radicals, reducing the production of inflammatory cytokines, regulating cell signaling pathways, inhibiting tyrosinase activity, and reducing the activity of enzymes that degrade the extracellular matrix. Furthermore, by-products of the agricultural and food industries, such as peels, seeds, leaves, and other plant residues, are considered valuable sources of natural bioactive compounds. Although these materials were previously regarded as waste, they often contain high amounts of bioactive compounds [5]. In addition to terrestrial plants, algae, fungi, microorganisms, and marine organisms are also valuable sources of bioactive substances owing to their unique chemical diversity and biological activities [6].

Even though many bioactive compounds have remarkable biological effects, most of them exhibit poor water solubility, low chemical stability, sensitivity to oxidation, photodegradation, limited skin penetration, and low bioavailability, making their direct use in cosmetic products difficult. In order to improve the stability, efficiency, and skin penetration of these bioactive compounds, a lot of research has been focused on developing green extraction, purification, and delivery techniques [7,8].

Previous reviews have focused on the use of natural products in cosmetic applications from different perspectives. Some have focused on specific plants, like *Nymphaea* spp. [9], *Punica granatum* [10], and *Dittrichia viscosa* [11], while others have explored topics such as green extraction methods and delivery systems, including nanoemulsions. In addition, many reviews have also been published on specific classes of natural compounds and their use in cosmetics [12,13,14] or have investigated specific applications, including anti-aging effects, antimicrobial properties and drug delivery systems [15,16,17]. Although these studies cover various aspects of natural compounds, further integrated analysis is needed to link their chemical classification, molecular mechanisms, extraction and purification methods, topical delivery, formulation stability, safety, and clinical evidence. Therefore, this review organizes natural bioactive compounds by chemical family and discusses their sources, cosmetic applications, extraction and purification methods, delivery systems, and formulation challenges. It also links chemical classification, biological activity, and formulation technologies to clarify both the potential benefits of natural bioactive compounds and the challenges involved in their use in cosmeceutical products.

## 2. Natural Bioactive Compounds in Cosmetics

In this review, natural bioactive compounds are grouped according to their chemical structure into flavonoids, terpenoids, phytosterols, nitrogen-containing compounds, fatty acids, vitamins, peptides and polysaccharides. The review focuses on compounds that occur naturally or are derived from natural sources.

### 2.1. Flavonoids and Their Derivatives

**Figure 1:**
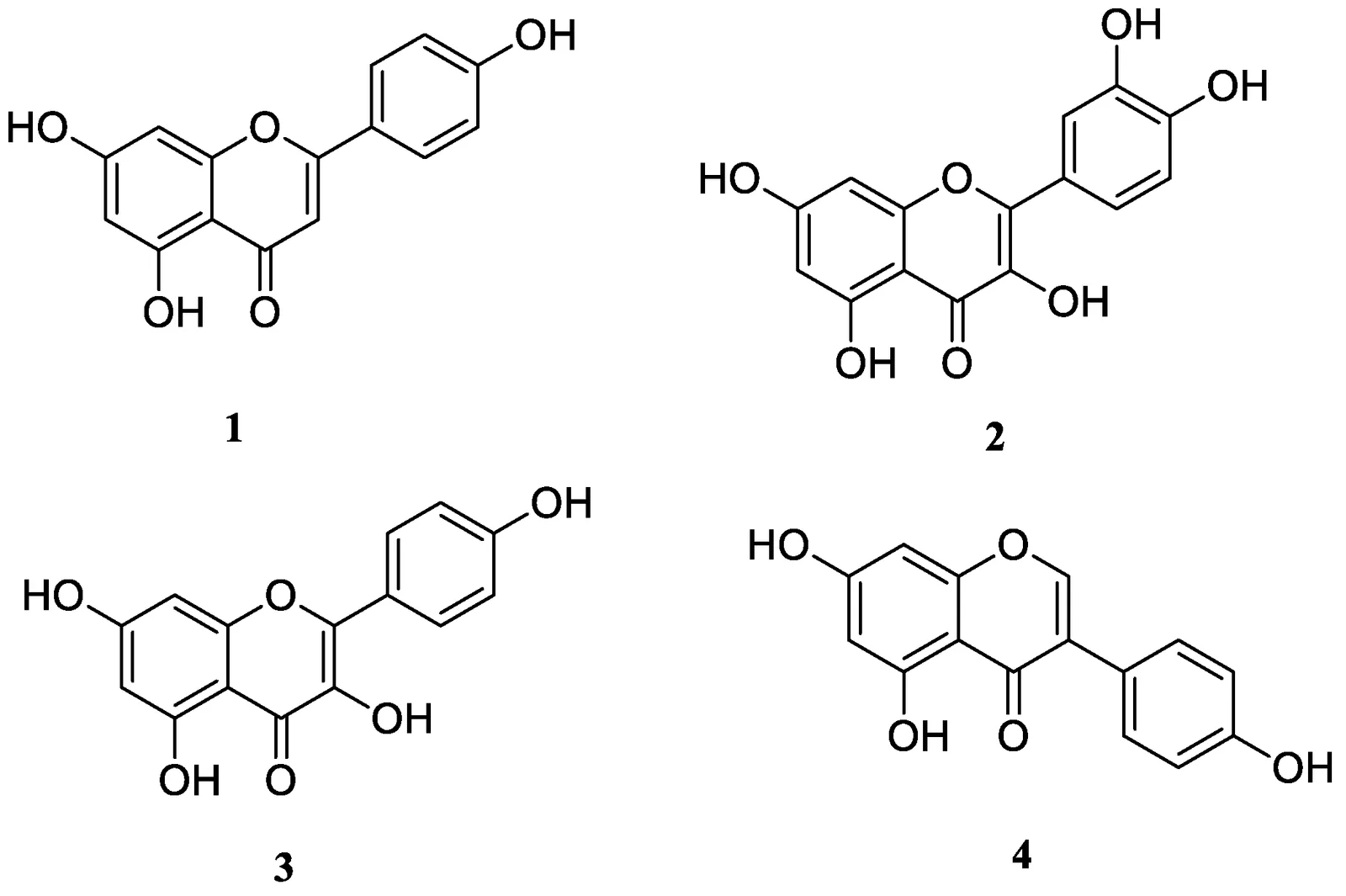
Chemical structures of apigenin (**1**), quercetin (**2**), kaempferol (**3**) and Genistein (**4**).

Flavonoids are one of the well-known natural bioactive compounds produced in different parts of plants as secondary metabolites [18]. Flavonoids are low-molecular-weight polyphenolic compounds mainly characterized by a 15-carbon skeleton (C_6_–C_3_–C_6_), which is composed of two phenyl rings and one heterocyclic ring [18,19]. Based on the degree of unsaturation and oxidation, flavonoids can be divided into several subclasses including flavones, flavonols, flavanones, flavan-3-ols (catechins), isoflavones, and anthocyanidins [20,21]. Among flavonoids, apigenin, quercetin, kaempferol, Genistein (Figure 1) and catechins have been frequently used in the cosmetic industry.

#### 2.1.1. Apigenin

Apigenin (5,7-dihydroxy-2-(4-hydroxyphenyl)-4*H*-1-benzopyran-4-one, compound **1**, Figure 1) is naturally presented in various plant species such as *Matricaria recutita* (chamomile), *Scutellaria barbata* (barbed skullcap), *Origanum vulgare* (oregano), *Ocimum basilicum* (basil), *Petroselinum crispum* (parsley), *Apium graveolens* (celery) and *Allium cepa* (onion), as well as in fruits, including *Carica papaya* (papaya), *Mangifera indica* (mango), *Fragaria* × *ananassa* (strawberry) and *Citrus sinensis* (orange) [22]. This bioactive compound demonstrates a wide range of biological activities relevant to skin health and therapeutic applications. The antioxidant properties are shown by the reduction in Reactive Oxygen Species (ROS), inhibition of the release of arachidonic acid from membrane lipids, and reduction in the MDA level. With regard to the anti-inflammatory effect, it reduces pro-inflammatory cytokines (Tumor Necrosis Factor Alpha (TNF-α), IL-6, IL-1β), blocks enzymes like COX-2 and iNOS, and lowers ROS formation. Also, it can inhibit melanocyte activity, and hence can be used in skin-brightening and anti-hyperpigmentation products. Additionally, it is utilized in anti-aging products [23], and is mainly integrated into serums, toners, facial masks, shampoos, and conditioners. Furthermore, this flavone is often combined with vitamin C and B-complex vitamins or essential oils in order to enhance product stability [7]. Although apigenin has low toxicity, high doses may lead to potential side effects, including muscle relaxation, upset stomach, skin irritation, and a mild sedative effect [24].

#### 2.1.2. Quercetin

Quercetin (2-(3,4-dihydroxyphenyl)-3,5,7-trihydroxy-4*H*-1-benzopyran-4-one, compound **2**, Figure 1) is a compound belonging to the group of flavonoids, and is an effective polyphenolic compound with high antioxidant and anti-inflammatory properties, which is widely found in fruits, vegetables, and food sources, including *Allium cepa* (red onion), *Malus domestica* (apples), *Brassica oleracea* (broccoli), *Solanum lycopersicum* (tomatoes), *Lactuca sativa* (lettuce), *Capsicum annuum* (chili peppers), *Vaccinium oxycoccus* (cranberries), *Rubus fruticosus* (blackberries), *Vaccinium corymbosum* (blueberries), *Capparis spinosa* (capers) and some other edible plants [25,26]. Quercetin plays an important role in the protection of the skin against photoaging, inflammation and hyper-pigmentation, through the neutralization of ROS, inhibition of the MAPK pathway, reduction in MMP-1 expression and inhibition of tyrosinase. Moreover, this substance shows good practicality in delivering when combined with vitamin C, niacinamide, and hyaluronic acid, which can even provide synergistic effects in enhancing antioxidant activity, hydrating, and improving the skin barrier [27]. Also, mixing quercetin with zinc can be used as sunscreen lipstick [28]. In addition, it can be used in peel-off masks with combinations of different polymers [25].

#### 2.1.3. Catechins

**Figure 2:**
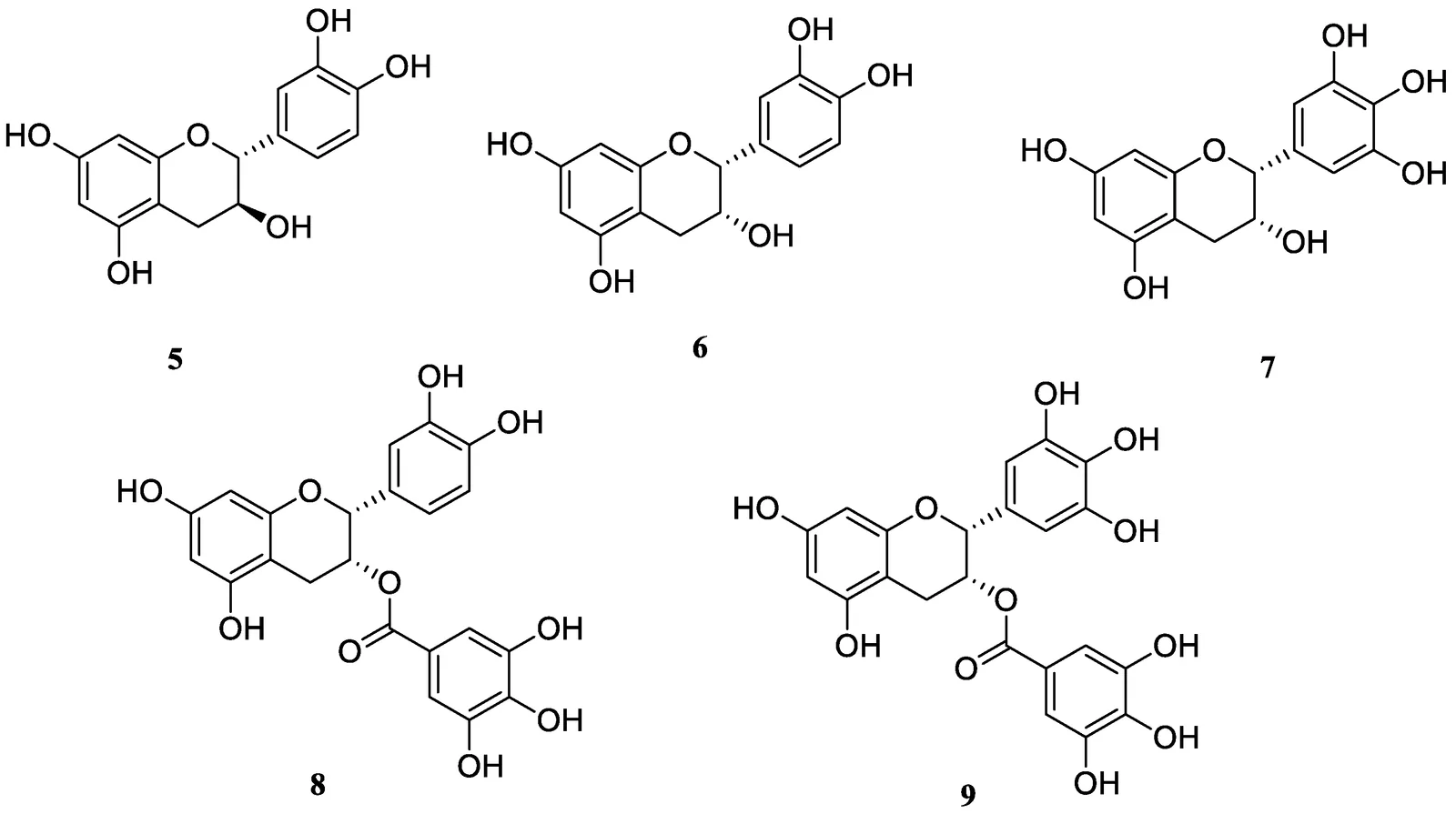
Chemical structures of (+)-catechin (C, **5**), (−)-epicatechin (EC, **6**), (−)-epigallocatechin (EGC, **7**), (−)-epicatechin gallate (ECG, **8**), and (−)-epigallocatechin gallate (EGCG, **9**).

Catechins are among the most important natural polyphenolic compounds that are generally found in *Camellia sinensis* (green tea) and *Canarium patentinervium* [29]. These compounds can also be found in other plant sources, including *Uncaria gambir* Roxb., *Canarium patentinervium*, grapes, apples, and pears. The most important catechin derivatives include (+)-catechin (C), (−)-epicatechin (EC), (−)-epigallocatechin (EGC), (−)-epicatechin gallate (ECG), and (−)-epigallocatechin gallate (EGCG) (compounds **5**–**9**, Figure 2), with EGCG being the most abundant and bioactive compound in green tea [30].

Catechins show several biological activities such as antioxidant, anti-inflammatory, antibacterial and UV protection properties [31]. These compounds delay skin aging by eliminating ROS, inhibiting inflammatory pathways such as NF-κB. In addition, they reduce the expression of matrix metalloproteinases (MMPs), prevent collagen degradation, and decrease the production of inflammatory mediators [30,32]. They have significant antibacterial activity by destroying bacterial cell membranes, inhibiting biofilm formation, reducing antibiotic resistance and producing a synergistic effect with some antibiotics [29]. Because of these bioactivities, catechins have been used in cosmeceutical products such as anti-aging creams and serums, sunscreens, anti-acne products, skin lighteners, toner, wound repair agents, shampoo, and mouthwashes [31,33]. Additionally, catechins have also been considered as natural preservative in cosmetic formulations due to their antibacterial and antifungal activities [29].

#### 2.1.4. Kaempferol

Kaempferol (3,5,7-trihydroxy-2-(4-hydroxyphenyl)-4*H*-1-benzopyran-4-one, compound **3**, Figure 1) is one of the most important natural flavonoids from the flavonol group [34]. This compound is widely found in various plant sources, including *Malus domestica* (apples), *Prunus armeniaca* (apricots), *Vitis vinifera* (grapes), *Prunus persica* (peaches), *Citrus sinensis* (oranges), *Actinidia deliciosa* (kiwi), *Brassica oleracea* var. *italica* (broccoli), *Brassica oleracea* var. *sabellica* (kale), *Spinacia oleracea* (spinach), *Allium cepa* (onions), *Asparagus officinalis* (asparagus), *Camellia sinensis* (green tea), and many medicinal plants such as *Aloe vera*, *Centella asiatica*, *Angelica keiskei*, *Cassia alata*, *Chromolaena odorata*, and *Capparis spinosa* [35,36]. Given its polyphenolic structure and the presence of four phenolic hydroxyl groups, kaempferol has significant anti-oxidant activity and protects cells and tissues by neutralizing free radicals and reducing oxidative stress. Studies have shown that kaempferol and its glycoside derivatives have a high ability to inhibit 2,2-diphenyl-1-picrylhydrazyl (DPPH) and 2,2′-azino-bis (3-ethylbenzothiazoline-6-sulfonic acid) (ABTS) free radicals, and play a role in controlling inflammation by reducing the expression of inflammatory and oxidative factors such as TNF-α, IL-6, TGF-β, HO-1 and NOX2 [34,37]. Kaempferol has numerous biological activities including antimicrobial, antidiabetic, cardiovascular and anticancer effects. Also, its ability to protect the skin against damage caused by ultraviolet (UV) radiation and reduce skin aging processes has made it a valuable compound in the formulation of cosmetic and health products such as anti-wrinkle creams, sunscreens and restorative lotions [38].

#### 2.1.5. Genistein

Genistein (5,7-dihydroxy-3-(4-hydroxyphenyl)-4*H*-1-benzopyran-4-one, compound **4**, Figure 1) represents one of the most important natural isoflavones found in legumes, especially *Glycine max* (soybeans). This compound is a bioactive phytoestrogen that exerts antioxidant, anti-inflammatory, and regenerative properties by binding to skin estrogen, in particular Estrogen receptor Beta (Erβ), which increases the proliferation of keratinocytes and fibroblasts [39,40]. The practicality of Genistein is observed in cosmetic products, wound healing, reducing oxidative stress, and maintaining hydration. Additionally, it decreases signs of skin aging by stimulating the proliferation of keratinocytes and fibroblasts, regulating the immune response, and increasing the synthesis of collagen and elastin [41,42]. Genistein components with bakuchiol, vitamins C, E and B3 and ceramides can produce synergistic effects in improving elasticity, hydration and skin tone and reducing wrinkles [43].

### 2.2. Terpenoids

Terpenoids are one of the largest and most diverse groups of natural secondary metabolites, consisting of isoprene (C_5_) units, and have attracted great attention in various fields, including the food, pharmaceutical, and cosmetic industries, due to their wide structural diversity and numerous biological activities. These compounds have diverse carbon skeletons that are divided into different groups, including hemiterpenes, monoterpenes, sesquiterpenes, diterpenes, sesterterpenes, triterpenes, and tetraterpenes, based on the number of isoprene units [44,45]. Terpenoids, including limonene, menthol, α-bisabolol, and glycyrrhizic acid, are widely recognized for their valuable applications in cosmetic formulations.

#### 2.2.1. Limonene

**Figure 3:**
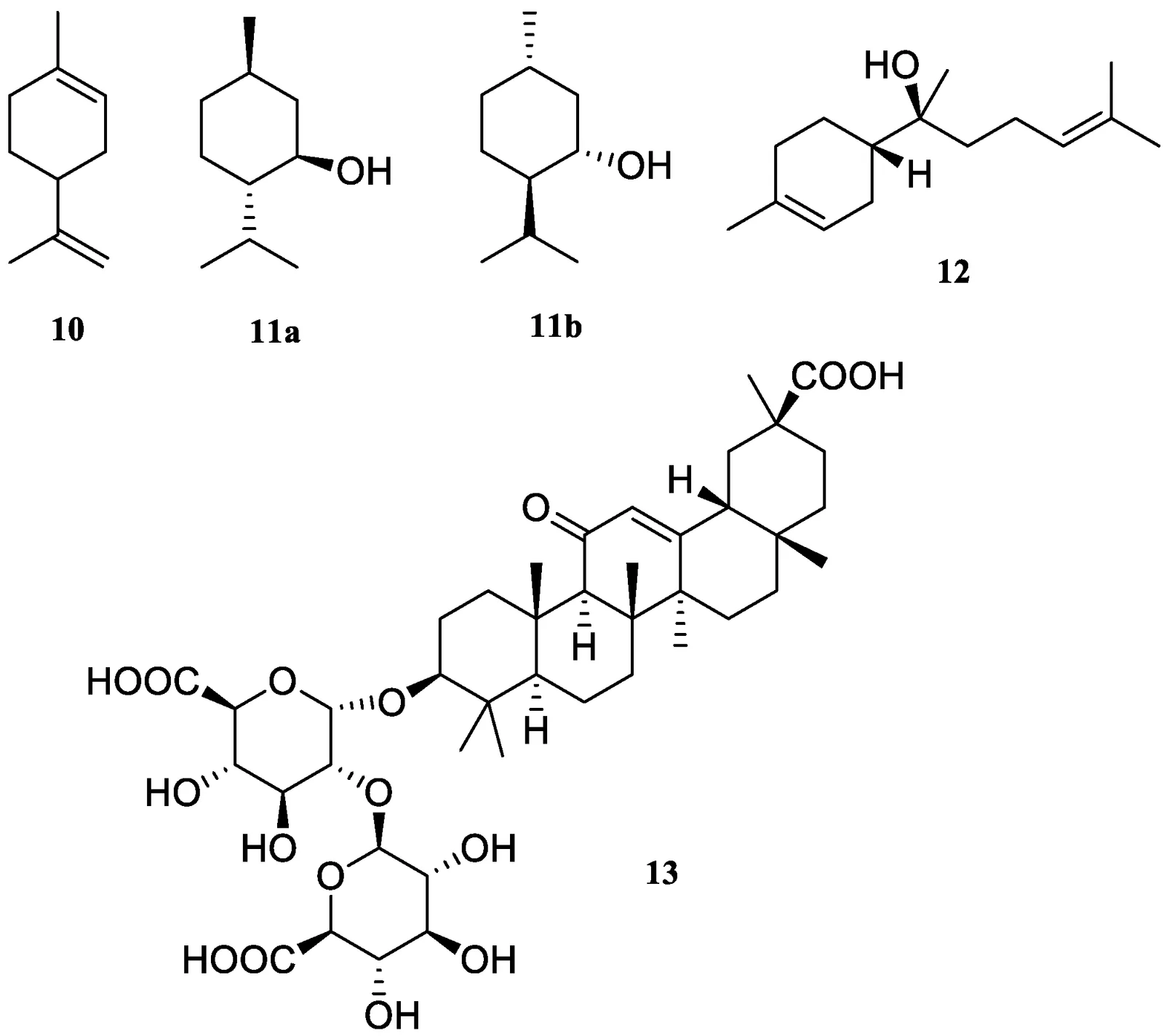
Chemical structures of limonene (**10**), (−)-and (+)-menthol (**11a** and **11b**), α-bisabolol (**12**), and glycyrrhizic acid (**13**).

One of the most important monocyclic monoterpene hydrocarbons is limonene (1-methyl-4-(prop-1-en-2-yl) cyclohex-1-ene) (compound **10**, Figure 3). This compound is present in citrus peels like, *Citrus sinensis* (orange) [46], *Citrus limon* (lemon) [47], *Citrus reticulata* (tangerine), and *Citrus paradisi* (grapefruit) with both enantiomeric forms (+)- and (−)-limonene [48]. Limonene has number of applications in the food, pharmaceutical, and cosmetic industries because of its favorable physicochemical properties, including lipophilicity, low toxicity, and biodegradability.

Limonene has antioxidant and anti-inflammatory properties; thus, it can slow down the production of inflammatory mediators (TNF-α, IL-6, IL-1β, NO and PGE2) by inhibiting inflammatory pathways such as NF-κB and MAPK and reducing the expression of iNOS and COX-2 [49,50]. It also protects skin cells from oxidative stress and DNA damage by reducing ROS and enhancing antioxidant enzymes (SOD, catalase and GPx) [50]. For these properties it is used as a fragrance, flavoring, natural solvent, and active ingredient in cosmetic formulations such as perfumes, soaps, shampoos, skin care formulations and anti-aging products [51]. However, limonene can be oxidized when exposed to air, and its oxidation products can act as sensitizers and trigger allergic contact dermatitis in susceptible individuals [52].

#### 2.2.2. Menthol

Menthol (2-isopropyl-5-methylcyclohexanol) is a cyclic monoterpene with two enantiomeric forms, (−)- and (+)-menthol (compounds **11a** and **11b**, respectively, Figure 3). Menthol and its components are extracted from various species of the genus Mentha, especially *Mentha* × *piperita* (peppermint), *Mentha arvensis*, and *Mentha canadensis* [53]. *Mentha* × *piperita* typically produces (−)-menthol, with smaller amounts of the stereoisomers (+)-neomenthol, (+)-isomenthol, and (+)-neoisomenthol, covering four of the possible eight stereoisomers [21]. Menthol is widely used in the food, pharmaceutical, and cosmetic industries because of its aroma, flavor, cooling effect and soothing properties. In general menthol is used in skin and oral care products [53], gels, creams, shampoo, and topical analgesic products. However, cutaneous irritation and contact sensitization have been reported, although the available literature suggests that clinically relevant adverse skin reactions are relatively uncommon and their frequency is not fully established [54,55]. Additionally, it has anti-inflammatory, antibacterial, antioxidant, antipruritic, analgesic, and skin-healing effects. Its anti-inflammatory and analgesic properties are primarily attributed to its regulation of TRP channels, particularly TRPM8, TRPV1, and TRPA1, and its reduction of pro-inflammatory cytokines like TNF-α and IL-6. It also prevents oxidative damage by reducing ROS and strengthening antioxidant systems. The lipophilic nature of menthol allows it to interact with the cell membrane of microorganisms and destroy it, giving it antibacterial and antifungal properties. Activation of TRPM8 causes a cooling sensation and reduces the transmission of itching and pain messages, so it can be used in skin care products, antipruritic and topical analgesic formulations [56]. Also, excessive consumption of menthol may cause skin irritation, allergic reactions, or gastrointestinal problems, so it is essential to use the appropriate concentration in formulations [53].

#### 2.2.3. α-Bisabolol

α-Bisabolol (α,4-dimethyl-α-(4-methyl-3-pentenyl)-3-cyclohexene-1-methanol, compound **12**, Figure 3) is a natural monocyclic sesquiterpene alcohol, which is extracted from *Matricaria recutita* (German chamomile) and *Eremanthus erythropappus* (Candeia). This compound has a variety of benefits not only in the pharmaceutical industry but also in the cosmetic industry because of its properties like low toxicity, no skin irritation or photosensitivity, as well as anti-inflammatory, antioxidant, antimicrobial, antifungal, and wound-healing properties [57]. Therefore, α-bisabolol has been extensively used in cosmetic products, such as creams, lotions, balms, moisturizers, anti-aging products, skin-brightening formulations, sunscreens, and especially skin repair products [58]. One of the most important biological properties of α-bisabolol is its antioxidant activity. This compound protects cells against ROS and Reactive Nitrogen Species (RNS), inhibiting lipid peroxidation, and increasing the activity of antioxidant enzymes such as superoxide dismutase (SOD), catalase (CAT) and glutathione (GSH). The anti-inflammatory activity of α-bisabolol is mainly applied through inhibition of NF-κB and MAPK pathways and reduction in expression of inflammatory mediators such as TNF-α, IL-1β, IL-6, COX-2, iNOS and PGE_2_. These effects decrease inflammation, preserve the extracellular matrix, and accelerate wound healing. In addition, α-bisabolol shows antibacterial and antifungal activities by preventing the growth of bacteria and fungi and increasing the effectiveness of some antibiotics through its ability to damage the cell membranes of microorganisms, inhibit the synthesis of ergosterol in fungi, and reduce drug resistance [59].

#### 2.2.4. Glycyrrhizic Acid

Glycyrrhizic acid (GL) (compound **13**, Figure 3) is one of the major bioactive compounds found in the roots of the *Glycyrrhiza glabra* (licorice plant) [60]. It consists of one molecule of glycyrrhetinic acid with hydrophobic properties, which is linked to two hydrophilic molecules of glucuronic acid [61,62]. This compound is widely used in the cosmetic, skin care, food, and pharmaceutical industries because of its biocompatibility, low toxicity, and wide range of biological activities. Its biological activities include anti-inflammatory, antioxidant, anti-allergic, antiviral, antibacterial, immunomodulatory, hepatoprotective, and antitumor properties, making it a valuable ingredient in cosmetic formulations [63]. Studies have shown that GL inhibits the HMGB1, NF-κB, NLRP3, and MAPK inflammatory signaling pathways while activating the nuclear factor erythroid 2-related factor 2/heme oxygenase-1 (Nrf2/HO-1) antioxidant pathway, thereby reducing the production of inflammatory mediators and ROS and preventing the degradation of the skin extracellular matrix [64,65]. These properties have led to the use of GL in the formulation of anti-aging and skin-brightening creams, anti-inflammatory products, wound-healing products, anti-acne products, and UV protection products [63].

### 2.3. Phytosterols

**Figure 4:**
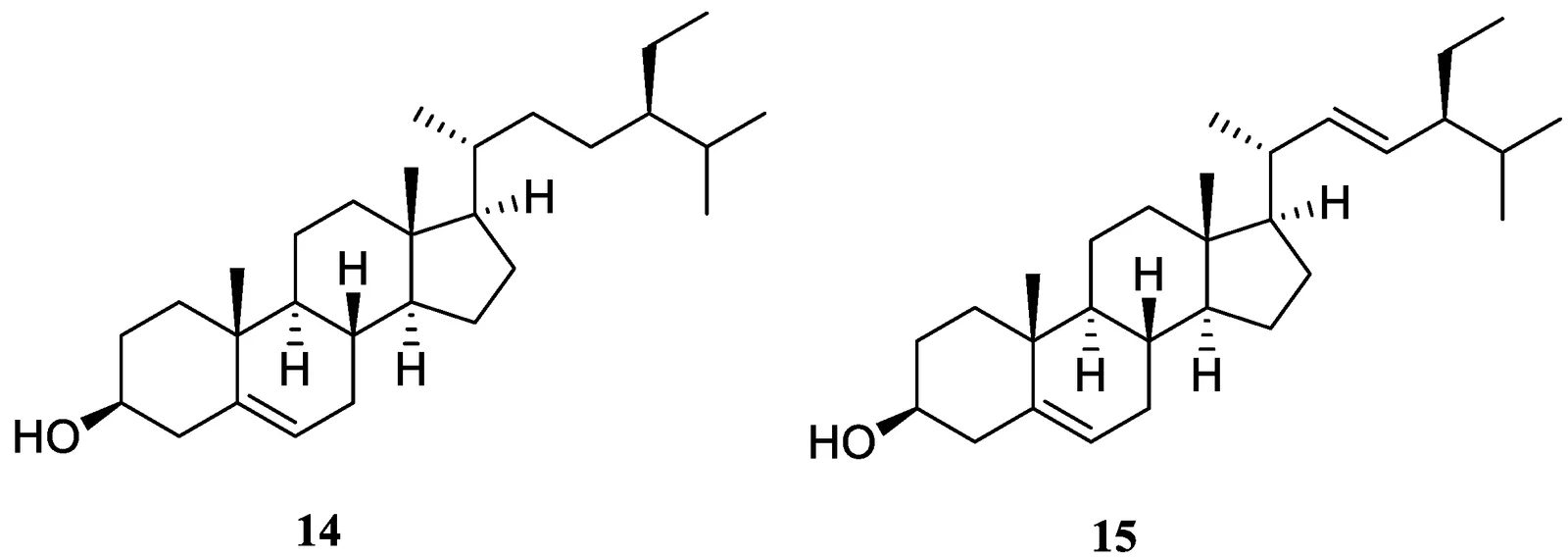
Chemical structures of β-sitosterol (**14**), and stigmasterol (**15**).

Phytosterols (plant sterols) are a group of sterols and important components of plant cell membranes. These compounds are derived from triterpenes and play an important role in maintaining membrane stability and function. The structure of these compounds is similar to cholesterol but they exhibit different biological properties because of the presence of an additional methyl or ethyl group at the C-24 position. Most phytosterols contain 28 or 29 carbon atoms and are unsaturated [66,67]. β-sitosterol and stigmasterol (compounds **14** and **15**, Figure 4) are two important phytosterols with potential applications in the cosmetic industry due to their diverse biological activities.

#### 2.3.1. β-Sitosterol

β-sitosterol ((1*R*,3a*S*,3b*S*,7*S*,9a*R*,9b*S*,11a*R*)-1-[(2*R*,5*R*)-5-ethyl-6-methylheptan-2-yl]-9a,11a-dimethyl-2,3,3a,3b,4,6,7,8,9,9a,9b,10,11,11a-tetradecahydro-1*H*-cyclopenta[a]phenanthren-7-ol, compound **14**, Figure 4) is one of the most common natural phytosterols in various parts of plants, including leaves, roots, rhizomes, fruits, and medicinal plants such as *Mirabilis jalapa* (four o’clock flower), and *Cucurbita pepo*) pumpkin). This phytosterol has a chemical structure similar to both cholesterol and steroid hormones, so it can interact with steroid receptors and take part in a wide range of biological activities [68]. It has anti-inflammatory, antimicrobial, antioxidant, antidiabetic, cholesterol-lowering, immune system-modulating, antiviral and anticancer properties [69]. The anti-inflammatory effects of β-sitosterol are mainly mediated through selective inhibition of cyclooxygenase-2 (COX-2), reducing the messenger RNA (mRNA) expression of this enzyme and decreasing the production of inflammatory mediators such as inter-leukin-6 (IL-6) and inducible nitric oxide synthase (iNOS). Because of its emollient and anti-inflammatory properties, β-sitosterol is utilized in the formulation of skin care products, including moisturizers, sunscreens, and anti-aging products, and its therapeutic potential in atopic dermatitis has also been reported [70].

#### 2.3.2. Stigmasterol

Stigmasterol ((1*R*,3a*S*,3b*S*,7*S*,9a*R*,9b*S*,11a*R*)-1-[(2*R*,3*E*,5*S*)-5-ethyl-6-methylhept-3-en-2-yl]-9a,11a-dimethyl-2,3,3a,3b,4,6,7,8,9,9a,9b,10,11,11a-tetradecahydro-1*H*-cyclopenta[a]phenanthren-7-ol, compound **15**, Figure 4) is another plant sterol which has a chemical structure very similar to β-sitosterol, differing only by the presence of an additional double bond in its side chain. It is widely found in vegetable oils, seeds, grains, legumes, vegetables, medicinal plants, and some microalgae. Because of its antioxidant, anti-inflammatory, antimicrobial, and immunomodulatory properties, stigmasterol has attracted increasing interest as a potential ingredient for skin care and cosmeceutical formulations [71]. Additionally, stigmasterol exhibits anti-inflammatory activity by reducing oxidative stress, strengthening the antioxidant defense system, and inhibiting the NF-κB and p38 MAPK inflammatory pathways. In addition, stigmasterol has shown antimicrobial activity against several Gram-positive and Gram-negative bacteria by inhibiting the growth of pathogenic microorganisms [71,72]. Recent studies have formulated stigmasterol in combination with β-sitosterol as topical gel–creams and films to improve skin delivery, highlighting its potential for topical skin care applications [67].

### 2.4. Nitrogen-Containing Compounds

**Figure 5:**
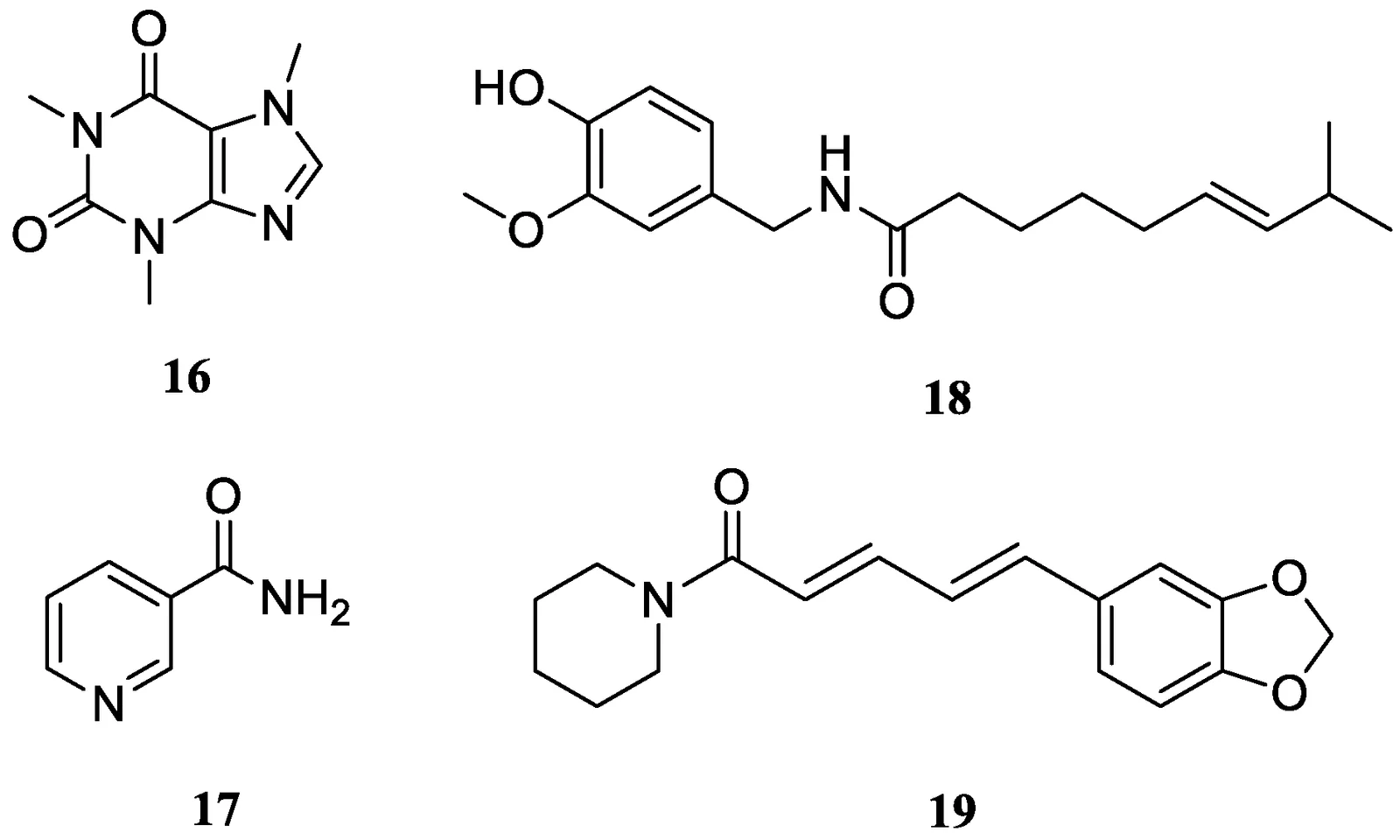
Chemical structures of caffeine (**16**), nicotinamide (**17**), capsaicin (**18**), and piperine (**19**).

Nitrogen-containing compounds are one of the important groups of plant secondary metabolites, which includes subsets of alkaloids, cyanogenic glycosides and glucosinolates. These compounds are generally obtained from amino acids and play an important role in plant defense against animals, pathogens and environmental stresses. These compounds have a wide range of biological activities, including antimicrobial, antiviral, anti-inflammatory, antioxidant and anticancer properties, so they are widely used in the pharmaceutical, food and cosmetic industries [73]. Caffeine, capsaicin and piperine are naturally occurring plant secondary metabolites, although caffeine is also produced industrially. Nicotinamide, the amide form of vitamin B3, is not a secondary metabolite and is also produced industrially [74]. These compounds (Figure 5) are examples of nitrogen-containing compounds used in cosmetic formulations because of their diverse biological activities and skin-beneficial properties.

#### 2.4.1. Caffeine

Caffeine (1,3,7-trimethylpurine-2,6-dione, compound **16**, Figure 5) is a well-known natural bioactive compound that is present as a secondary metabolite in more than 60 plant species. It comes from *Coffea arabica* and *Coffea canephora* (coffee beans), *Camellia sinensis* (tea leaves), *Theobroma cacao* (cocoa beans), *Cola acuminata* (cola seeds), and *Paullinia cupana* (guarana). This compound is not only extracted from natural sources but also produced industrially [75]. It is a white, odorless, bitter-tasting powder that is soluble in both water and fat. Caffeine can be obtained naturally from plant leaves, seeds, and fruits through various extraction methods. This compound is extensively utilized in cosmetic products such as anti-aging creams, anti-cellulite products, eye creams, scrubs, facial serums, facewash, face mask lipsticks and shampoos owing to its antioxidant, anti-aging, anti-inflammatory, anti-cellulite, UV protection, microcirculation improvement, and hair growth stimulation properties. Caffeine works by inhibiting the phosphodiesterase enzyme (PDE), increasing cAMP levels, lipolysis, blood microcirculation and lymphatic drainage and helping to reduce cellulite and edema [76,77]. It also protects against photoaging and UV damage by suppressing collagenase and elastase enzymes and decreasing oxidative stress by inhibiting lipid peroxidation and eliminating free radicals [76,77].

#### 2.4.2. Nicotinamide

Niacinamide (pyridine-3-carboxamide; 3-pyridinecarboxamide, compound **17**, Figure 5) is the amide form of vitamin B3 and the main precursor of nicotinamide adenine dinucleotide (NAD+), which plays a key role in cellular energy production, DNA repair, regulation of redox balance, and maintenance of skin cell function [78]. This compound applies its biological effects by increasing intracellular NAD+ levels and activating NAD+-dependent enzymes like SIRT1, SIRT3, and SIRT6. This increase promotes the production of adenosine triphosphate (ATP), enhances DNA repair, improves mitochondrial function, reduces oxidative stress, and inhibits cellular aging. Its anti-inflammatory effects are mediated by inhibiting the NF-κB pathway and reducing the expression of inflammatory cytokines, thus helping to control acne, rosacea, and other inflammatory skin diseases [79]. Niacinamide can improve the skin’s barrier function by increasing the synthesis of ceramides, cholesterol, and free fatty acids, which decrease TEWL and improve skin moisture. In addition, it also reduces hyperpigmentation and uniform skin tone by inhibiting the transfer of melanosomes [80]. Niacinamide not only inhibits skin aging by reducing ROS and enhancing the repair of UV-induced damage but also reduces wrinkles and improves skin firmness and elasticity by stimulating collagen and elastin synthesis and inhibiting matrix metalloproteinases (MMPs) [79]. Because of these advantages, niacinamide is widely used in cosmetic products such as facial skin-lightening creams, underarm skin-lightening products, anti-hair loss products, sunscreens, moisturizing products, anti-aging creams, and skin-brightening creams [74,81].

#### 2.4.3. Capsaicin

Capsaicin (trans-8-methyl-*N*-vanillyl-6-nonenamide, compound **18**, Figure 5) is the most important member of the capsaicinoid family and one of the main bioactive compounds found in the fruits of *Capsicum annuum* (chili pepper) [82,83]. This compound is highly lipophilic, so it penetrates the skin’s lipid barrier, making it a suitable candidate for topical formulations. This compound has many biological activities, including anti-inflammatory, antioxidant, antimicrobial, and analgesic properties, which make it one of the most important bioactive compounds used in cosmetic and skin care products. Capsaicin inhibits the transmission of pain and itching signals through the activation of the TRPV1 receptor in sensory nerve terminals by increasing the influx of calcium and sodium ions, thereby inducing the release of neuropeptides such as substance P and calcitonin gene-related peptide (CGRP). It also exhibits antioxidant activity by activating the nuclear factor erythroid 2-related factor 2/Antioxidant Response Element (Nrf2/ARE) signaling pathway, enhancing the expression of endogenous antioxidant enzymes and reducing ROS. Its anti-inflammatory effects are mediated by inhibiting the NF-κB and MAPK inflammatory pathways, activating PPARγ, and reducing the expression of inflammatory cytokines, including TNF-α, IL-1β, IL-6, and IL-8, as well as COX-2 and iNOS [84]. Additionally, capsaicin exhibits antimicrobial activity by disrupting the membranes of microorganisms and increasing their permeability. Capsaicin also increases skin blood flow through the activation of TRPV1, making it a useful compound in volumizing lipsticks and lip glosses, hair growth-stimulating products, sun protection products, anti-aging products, and products for soothing sensitive skin [82,85]. However, its topical use may cause local skin irritation, including burning, erythema, and discomfort, depending partly on concentration and exposure conditions [86].

#### 2.4.4. Piperine

Piperine ((2*E*,4*E*)-5-(1,3-benzodioxol-5-yl)-1-(piperidin-1-yl) penta-2,4-dien-1-one, compound **19**, Figure 5) is an amide alkaloid that can be extracted from *Piper nigrum* L. (black pepper) and *Piper longum* L. (long pepper) [85,87]. The most important characteristics of piperine for cosmetic applications are its anti-inflammatory, antioxidant, skin penetration-enhancing, and melanogenesis-stimulating properties [88]. Additionally, piperine has the ability to control inflammation and improve skin diseases such as dermatitis and psoriasis by inhibiting the NF-κB, MAPK, and STAT inflammatory pathways and reducing the production of inflammatory mediators, including TNF-α, IL-1β, IL-6, COX-2, and iNOS. Moreover, it protects skin cells from oxidative stress and ultraviolet-induced aging by reducing ROS production, enhancing the activity of antioxidant enzymes like SOD, CAT, and glutathione peroxidase (GPx), and stimulating the Nrf2/HO-1 pathway [89,90]. Furthermore, it facilitates the absorption of simultaneously applied substances into the skin and promotes the formation of melanocytes and melanin, thereby becoming a potential ingredient in anti-aging and wound-healing products, treatments for depigmentation disorders, sunscreens, creams, lotions, and gels [85].

### 2.5. Fatty Acids

**Figure 6:**
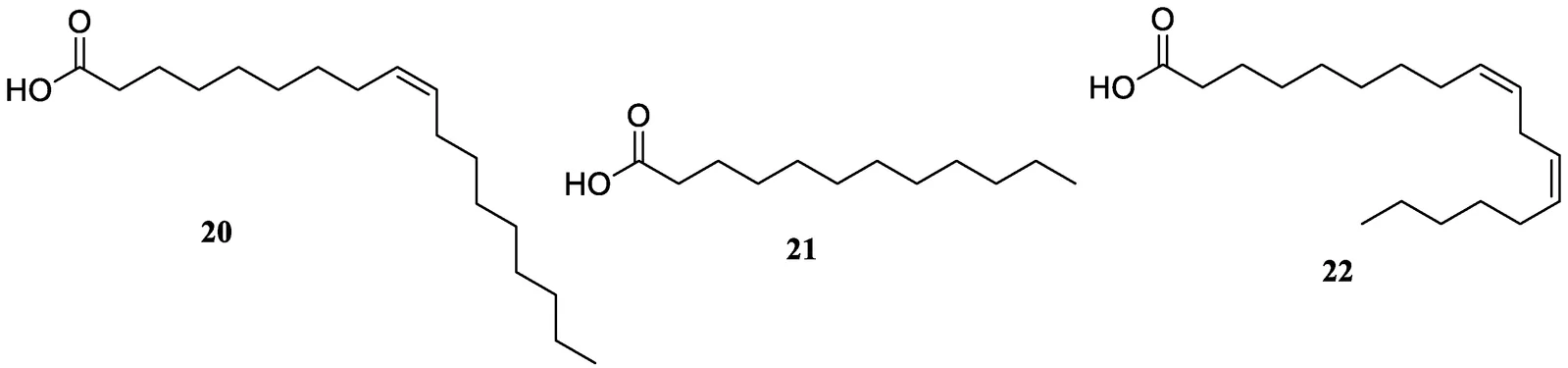
Chemical structures of oleic acid (**20**), lauric acid (**21**), and linoleic acid (**22**).

Fatty acids are one of the groups of natural lipid compounds with a hydrocarbon chain and a carboxyl group. These compounds are the principal constituents of vegetable oils and feature the ability to structure cell membranes and energy storage. They have valuable biological activities such as anti-inflammatory, antimicrobial, and antioxidant properties. Owing to these properties, fatty acids have a wide range of applications in the food, pharmaceutical, and cosmetic industries [91]. Compounds such as oleic acid, lauric acid, and linoleic acid (Figure 6) have also been reported for their potential use in the cosmetic industry.

#### 2.5.1. Oleic Acid

Oleic acid ((*Z*)-octadec-9-enoic acid, compound **20**, Figure 6) is a monounsaturated fatty acid from the omega-9 group that is prevalent in vegetable oils and butters [92]. Virgin olive oil (VOO) is one of the richest natural sources of oleic acid, and lots of biological properties and applications of this oil are connected with the oleic acid [93]. The most significant property of oleic acid is skin penetration enhancement. Oleic acid reacts with skin lipids, changes their organization, decreases their density and thickness, causes phase separation and, consequently, facilitates the penetration of other active ingredients through the skin [92]. However, high amounts of oleic acid can disrupt the skin barrier, increase TEWL, and cause skin irritation [92,94]. Therefore, the choice of an appropriate amount and the development of optimal formulations is critical for obtaining the advantages of this fatty acid and avoiding its side effects.

#### 2.5.2. Lauric Acid

Lauric acid (Dodecanoic acid, compound **21**, Figure 6) is one of the most famous medium-chain fatty acids generally found in both plant and animal sources. *Cocos nucifera* (coconut) oil and palm kernel oil are the most important natural sources of lauric acid [95]. In addition, it is also found in human milk, cow’s milk, goat’s milk, some plant species, and oil extracted from the larvae of *Hermetia illucens* (black soldier fly; BSF). BSF contains a high amount of lauric acid, ranging from approximately 37% to 67%, depending on feeding and extraction conditions [96]. Therefore, it can be used as a sustainable and economical alternative source of lauric acid. Among fatty acids, lauric acid is considered one of the strongest antimicrobial fatty acids because it can inhibit the growth of a wide range of microorganisms by increasing the permeability and destabilization of cell membranes, disrupting the electron transport chain, inhibiting oxidative phosphorylation, and hindering membrane-associated enzymes and proteins. Furthermore, it not only exhibits high activity against Gram-positive bacteria but also shows effectiveness against Gram-negative bacteria in some cases, as well as antifungal, antiviral, and antioxidant activities [95,97]. Hence, lauric acid has been widely used in the cosmetic and healthcare industries. Lauric acid and its derivatives are among the most important raw materials for the production of surfactants, emulsifiers, and cleansing agents because they reduce surface tension, allow water to mix with oils and dirt, and improve the cleansing performance of cosmetic products. The most important derivatives of lauric acid used in the production of shampoos, body washes, liquid and transparent soaps, facial cleansers, creams, and lotions include sodium lauryl sulfate, sodium lauryl ether sulfate, methyl laurate, ethyl laurate, zinc laurate, and acyl glycinate surfactants. It has also been used in the formulation of skin care products to increase hydration, improve cell regeneration, and inhibit acne-causing bacteria [98,99].

#### 2.5.3. Linoleic Acid

Linoleic acid ((9*Z*,12*Z*)-octadeca-9,12-dienoic acid, compound **22**, Figure 6) is one of the most abundant unsaturated fatty acids in the epidermis and belongs to the omega-6 family. A fundamental role of linoleic acid is its ability to maintain skin homeostasis, preserve epidermal barrier integrity, and regulate inflammatory responses [100]. Since the human body is unable to synthesize this fatty acid, it must be obtained through food or topical application. The main sources of linoleic acid are vegetable oils such as *Carthamus tinctorius* (safflower), *Helianthus annuus* (sunflower), *Glycine max* (soybean), *Zea mays* (corn), *Sesamum indicum* (sesame), *Vitis vinifera* (grape seed) and *Oenothera biennis* (evening primrose) oils [101,102]. Linoleic acid plays a vital role in the synthesis of epidermal ceramides, especially esterified omega-hydroxyacyl sphingosine ceramide (CER[EOS]), as these ceramides are essential for reducing transepidermal water loss, maintaining skin moisture, and preventing the penetration of external factors [103]. Moreover, linoleic acid acts as a natural ligand for PPAR-α receptors, leading to increased keratinocyte differentiation, stimulation of epidermal lipid synthesis, improvement of skin barrier function, regulation of lipid metabolism, and reduction in inflammation. This compound also improves acne, dermatitis, and other skin disorders by inhibiting the production of pro-inflammatory cytokines, including TNF-α, IL-1β, and IL-6, and by modulating inflammatory responses. In addition, it reduces hyperpigmentation by inhibiting the activity of tyrosinase and TRP-2 and increasing the removal of melanin from the epidermis. Because of these properties, linoleic acid has recently been recognized as one of the most important active ingredients in cosmetic and skin care products [103]. Therefore, it is widely utilized in the formulation of moisturizers, skin barrier repair creams, anti-acne products, anti-inflammatory products, skin-lightening and anti-blemish products, wound-healing creams, lip balms, and anti-aging products [103,104].

### 2.6. Vitamins

Vitamins are organic compounds that are divided into two groups like water-soluble and fat-soluble. They play a fundamental role in regulating metabolic processes, maintaining health and body function. Among them, vitamins A, C, E and some of their analogs are the most widely used in topical formulations because of their important roles in collagen synthesis, cell regeneration, maintenance of skin moisture, and protection against oxidative stress [105,106].

#### 2.6.1. Vitamin A

Vitamin A comes from foods of animal origin like meat, milk and fish (retinol and retinyl esters) as well as from colorful fruits and vegetables (beta-carotene) [107]. This vitamin refers to a group of fat-soluble compounds, including retinol, retinal, retinoic acid (tretinoin), and retinyl esters, collectively known as retinoids [108]. Although retinoic acid is the most active compound in this family, for topical application, retinol and retinyl esters must first be converted to retinal and then to retinoic acid to exert their biological effects. The biological activity of retinoids follows the order retinoic acid > retinal > retinol > retinyl esters, whereas skin tolerance follows the reverse order [109]. Therefore, retinol and retinyl esters are the most commonly used retinoid derivatives in cosmetic products because of their lower irritancy and greater stability. Retinoids are among the most well-known active compounds in anti-aging skin care products. They regulate the expression of genes involved in cell growth and differentiation by binding to retinoic acid receptors (RARs) and retinoid X receptors (RXRs) [110]. Additionally, retinoids improve skin appearance by increasing keratinocyte proliferation and differentiation, stimulating fibroblast activity, enhancing collagen and glycosaminoglycan synthesis, inhibiting matrix metalloproteinases (MMPs), and reducing extracellular matrix degradation. Retinoic acid is regarded as the gold standard for the treatment of photoaging, but it is available only by prescription because of its side effects and the risk of teratogenicity. In contrast, both retinol and retinyl esters are widely used in cosmetic products because of their better tolerability. They can be used in serums, creams, moisturizers, lotions, hand and eye creams, facial masks, lipstick, make-up removers, shampoo and liquid hand soap [111,112].

#### 2.6.2. Vitamin C

Vitamin C is a water-soluble vitamin that exists in two forms: L-ascorbic acid and dehydroascorbic acid. The human body is unable to synthesize this vitamin, so it must be obtained through foods or topical formulations. The main sources of vitamin C include *Citrus* spp. (citrus fruits), *Actinidia deliciosa* (kiwi), *Fragaria* × *ananassa* (strawberry), *Psidium guajava* (guava), *Malpighia emarginata* (acerola), *Capsicum annuum* (pepper), *Brassica oleracea* var. *italica* (broccoli), *Spinacia oleracea* (spinach), *Solanum lycopersicum* (tomato), and other fresh fruits and vegetables [113,114]. Although it is found in epiderma, the amount of it is lower in old people and people with sun-damaged skin compared with young adults. Antioxidant properties are one of the valuable properties of vitamin C, because it prevents oxidative damage to cells by eliminating ROS and RNS caused by ultraviolet radiation and pollution, and enhances the protective effects of vitamin E by regenerating it [107,113,114]. Moreover, vitamin C prevents collagen degradation and photoaging of the skin by reducing MMPs. Not only does vitamin C improve skin elasticity and reduce wrinkles by increasing the expression of collagen types I and III, but it also helps treat hyperpigmentation by inhibiting tyrosinase activity and reducing melanin synthesis. It is utilized in facial masks, anti-aging and anti-wrinkle creams, brightening and anti-spot creams and serums [111,115].

#### 2.6.3. Vitamin E

Vitamin E is composed of eight isomers, four tocopherols (α, β, γ, and δ) and four tocotrienols (α, β, γ, and δ) which are fat-soluble compounds with excellent antioxidant properties [116]. Among these compounds, α-tocopherol is the most biologically active form in human tissues, especially the skin, while γ-tocopherol is the predominant form in the diet [117]. Natural sources of vitamin E include vegetable oils such as *Triticum aestivum* (wheat germ), *Helianthus annuus* (sunflower), *Carthamus tinctorius* (safflower), *Glycine max* (soybean), *Zea mays* (corn) and *Brassica napus* (canola) oils; nuts and seeds such as *Prunus dulcis* (almond), *Arachis hypogaea* (peanut), *Corylus avellana* (hazelnut) and sunflower seeds; and fruits and vegetables such as *Persea americana* (avocado), *Spinacia oleracea* (spinach), *Brassica oleracea* var. *italica* (broccoli), *Capsicum annuum* (red bell pepper), *Actinidia deliciosa* (kiwi) and *Mangifera indica* (mango) [107,118]. In cosmetic formulations, α-tocopherol and its ester derivatives, particularly α-tocopheryl acetate, are most commonly used because of their greater stability. These esterified forms are converted to biologically active α-tocopherol after penetrating the skin. By neutralizing ROS and inhibiting lipid peroxidation, vitamin E protects cell membranes, proteins, and DNA from oxidative damage [119]. It also improves skin health by reducing inflammation, strengthening the skin barrier, and maintaining skin moisture, and its antioxidant activity is enhanced in combination with vitamin C [116,119]. Because ultraviolet (UV) radiation and other environmental stressors deplete cutaneous vitamin E, it is widely used in many skin care, anti-aging, and hair growth products to protect against photoaging and oxidative skin damage [117,120].

### 2.7. Peptides with Cosmeceutical Activity

Peptides are short chains of amino acids with 2 to 50 amino acids in their structure. Due to their antioxidant, antimicrobial, and anti-inflammatory activity, good safety, low allergenicity, and cost-effectiveness, peptides are considered among the most important active ingredients in cosmetic and skin care formulations [121,122]. These compounds can be produced by chemical synthesis or extracted from natural sources such as plants like *Glycine max* (soybeans) and *Oryza sativa* (rice); animals (milk, eggs, and meat); marine organisms (algae, fish, sea sponges, and marine microorganisms); yeast (*Saccharomyces cerevisiae*); and edible insects. Synthetic and chemically modified peptides, such as palmitoylated and acetylated peptides, are beyond the scope of this review, which focuses on naturally derived peptides [123,124]. Among naturally derived peptides, soy peptides, rice peptides and silk fibroin are classified as enzyme-inhibiting peptides, which inhibit extracellular matrix-degrading enzymes, prevent collagen degradation, increase skin moisture, exhibit antioxidant activity and help reduce hyperpigmentation [124,125].

### 2.8. Polysaccharides with Cosmeceutical Activity

Natural polysaccharides are extracted from a variety of sources such as plants, algae, fungi, bacteria, and some animal sources [126]. Polysaccharides are considered important ingredients in the cosmetic and pharmaceutical industries because of their properties such as biocompatibility, biodegradability, less toxicity, and hydrogel and film formation ability. The physicochemical and biological properties of polysaccharides are influenced by several factors including molecular weight, monosaccharide composition, the type of glycosidic bond, the degree of branching, conformational structure, and the presence of functional groups such as sulfate, carboxyl, and uronic acid [126]. In cosmetic products, polysaccharides are generally classified into two groups. The first group, known as functional polysaccharides, is used as thickeners, gelling agents, emulsifiers, film formers, stabilizers, and softeners in formulations. The second group, known as bioactive polysaccharides, is not only used in cosmetic formulations but also exhibits moisturizing, antioxidant, anti-inflammatory, wound-healing, anti-aging, collagenase- and elastase-inhibitory, and melanogenesis-inhibitory activities. Furthermore, these properties play an important role in maintaining skin health. They help to accelerate wound healing and tissue regeneration through their antioxidant, anti-inflammatory, and film-forming properties. Their hydrogel-forming ability improves water retention in the stratum corneum, thereby reducing TEWL and increasing skin hydration. In addition, they delay the skin aging process by reducing ROS, inhibiting MMPs, protecting collagen from degradation, and stimulating collagen synthesis [126,127]. Table 1 shows the most important polysaccharides used in cosmetic and skin care products.

## 3. Challenges and Future Perspectives

### 3.1. Plant Harvesting and Quality Control

Chemical compounds extracted from plant species are influenced by many environmental parameters like geographical origin, climatic conditions, soil composition, plant genetics, harvest season and time, and extraction method. These changes can affect the phytochemical profile and concentration of bioactive compounds and cause variability between different batches. Quality control and standardization of plant extracts play an important role in maintaining the quality of plant products. For this reason, there are guidelines for Good Agricultural and Collection Practices (GACP), which include control of planting materials, proper soil management, optimization of cultivation conditions, and standardization of harvesting and processing steps. Despite many efforts, there are a variety of frameworks and standardization requirements in different countries and regions. In some Asian countries, the use of plant metabolites is based on historical usage and national policies, so the lack of a standardized and global framework can lead to inconsistencies in the quality and efficacy of plant products. Furthermore, the presence of heavy metals, pesticides, and adulterated substances can affect the quality and safety of plant bioactive compounds, increasing the need for more rigorous quality control [144,145].

### 3.2. Extraction and Purification Methods

Extraction of bioactive substances from plants depends on various factors which include the plant species, solvent polarity, temperature, pH, extraction time, and the physicochemical properties of the target compounds [146]. Although traditional extraction methods such as solvent extraction [147], maceration [148], steam distillation, Soxhlet extraction, cold pressing, and hydroalcoholic extraction are still widely used, new green extraction technologies are increasingly being developed. These techniques include ultrasound-assisted extraction (UAE), microwave-assisted extraction (MAE) [148], supercritical fluid extraction (SFE) [149], enzyme-assisted extraction, pulsed electric field, Ohmic heating [44], Pressurized Liquid Extraction (PLE) [93], and deep eutectic solvents [75,150]. These methods are different in extraction efficiency, target compounds, and solvent consumption, and the choice of method depends on the chemical class of the compound and the scale of production. Extraction methods also play a crucial role in determining the bioactivity and stability of the extracted compounds. After the extraction of different bioactive compounds, they are purified for utilization in the cosmetics and pharmaceuticals industries through several techniques, such as column chromatography, preparative high-performance liquid chromatography (HPLC), gas chromatography (GC), solid-phase extraction, crystallization and distillation methods. In addition, analytical techniques such as HPLC, LC-MS and GC-MS are important for the characterization and quality control of botanical extracts, which usually contain complex mixtures of bioactive compounds [84].

### 3.3. Drug Delivery Systems for Plant-Derived Bioactive Compounds

Plant-derived bioactive compounds show significant biological activities for cosmetic and therapeutic applications but many of them have limitations like poor water solubility, low bioavailability [7], high chemical instability [7], rapid degradation [8], high volatility, and limited skin penetration [151,152]. These limitations reduce their stability, efficacy, and dermal absorption in topical formulations. Moreover, the formulation of natural compounds is according to their physicochemical properties such as structure, molecular size, polarity and solubility, which determine whether they can reach the target skin layer. For example, vitamin C is water-soluble but sensitive to oxidation, and its poor lipid solubility limits its penetration through the skin barrier. In contrast, lipophilic compounds such as vitamin E are more compatible with oil-based formulations. Capsaicin can penetrate the lipid barrier of the skin more easily, and oleic acid acts as a penetration enhancer for other active ingredients by changing the organization of skin lipids. Polyphenols, such as quercetin, may have limited water solubility. Polysaccharides are large molecules and their properties depend on their molecular weight and structure, and they mainly improve skin hydration by forming a film that retains water in the stratum corneum. The pH of the formulation, temperature, storage conditions, and interactions with excipients may also affect stability and performance. In order to overcome these challenges, various advanced drug delivery systems have been developed, including liposomes, nanoemulsions, microemulsions, niosomes, polymeric nanoparticles, nanocapsules, solid lipid nanoparticles (SLNs), nanostructured lipid carriers (NLCs), micelles, microneedles, and other nanocarrier-based systems [153,154]. These delivery platforms enhance the stability, bioavailability, skin penetration, controlled release, and overall therapeutic performance of bioactive compounds, improving their effectiveness in cosmetic and pharmaceutical formulations.

### 3.4. Safety, Toxicity and Regulatory Considerations

Although bioactive compounds extracted from plants have a natural origin, they are not always risk-free and can lead to dose-dependent effects, toxicity, adverse reactions, skin sensitization, and plant–drug interactions. Some plant-derived compounds have beneficial effects at low concentrations, but higher concentrations or continuous skin exposure can cause toxicity, irritation, allergic contact dermatitis, and phototoxicity. In addition, some plant compounds may have potential endocrine-disrupting effects, raising concerns about the safety of long-term use. Some of them can affect CYP450 enzymes and metabolic transporters and alter the efficacy or toxicity of concomitant medications. Therefore, safety assessment should include chemical characterization, exposure assessment on the skin, toxicity and sensitization for skin care products, and control of contaminants such as heavy metals and pesticides [145,155]. Toxicological findings from oral administration should not be directly used for topical applications, because differences in absorption and internal exposure between these routes must be considered in cosmetic safety assessment [156]. It should be noted that the term “cosmeceutical” is not defined as a separate regulatory category. The European Union, the United States, Canada, Japan, and China differ in their approaches to product classification, ingredient control, safety assessment, and labeling requirements. For example, in the USA and Canada, products with sunscreen or anti-dandruff claims may be regulated as drugs, Japan has a separate category called quasi-drugs, and China classifies whitening and sunscreen products as special cosmetics that require registration before marketing. In general, the classification of products depends on their composition, intended use, and claimed effects. Therefore, ingredient traceability, manufacturing quality, toxicological evaluation, and scientific substantiation of cosmetic claims are essential for product safety and regulatory compliance [157].

### 3.5. Molecular Mechanisms and Their Relevance to Cosmetic Outcomes

Many of the natural bioactive compounds discussed in Section 2 have been reported to modulate pathways involved in oxidative stress (ROS scavenging and Nrf2/ARE-dependent antioxidant enzymes like HO-1, CAT and SOD), inflammation (NF-κB, MAPK, COX-2 and TNF-α), extracellular matrix turnover (MMP-1 and collagen synthesis), melanogenesis (tyrosinase activity and melanosome transfer) and ceramide synthesis. These mechanisms provide reasonable grounds for the use of natural compounds in cosmetic products. For example, reduction in NF-κB/MAPK signaling and lower ROS levels in UV-exposed keratinocytes or fibroblasts are expected to limit MMP-1 induction and collagen degradation, which can delay skin aging, because degradation of type I and type III collagen leads to a wrinkled appearance and reduced skin elasticity [158]. In addition, UV exposure disrupts the function of the skin barrier by altering stratum corneum lipids and increasing TEWL. Therefore, increased ceramide synthesis is relevant to barrier function when it is reflected in lower TEWL and higher skin hydration. Similarly, inhibition of tyrosinase or melanosome transfer is relevant to pigmentation when it reduces the melanin index or visible pigmentation, and anti-inflammatory and antioxidant activities can be related to reduced UV-induced erythema. For many plant-derived ingredients, the available data derive from in vitro and animal studies only, and cosmetic benefits supported only by mechanistic data should therefore be regarded as potential rather than demonstrated [159,160].

### 3.6. Clinical Validation

Despite the promising results of laboratory studies, translating these findings into clinical efficacy remains challenging. Effective concentrations of plant materials which are obtained from laboratory studies may not be achievable in humans. Additionally, differences in skin structure, metabolism, and immune responses in animal models limit their ability to predict human outcomes. Human clinical evidence for many plant bioactive compounds remains limited, and many studies have been conducted with small numbers of participants, short durations, and endpoints that may not fully reflect true clinical improvement. Therefore, the gap between biological activity observed in in vitro studies and clinical outcomes remains a major challenge in the development of these compounds [161,162]. An important point to consider is the safety of the concentrations tested and the experimental models used, since these characteristics influence the relevance of the findings to topical cosmetic applications. A recent review of coffee-derived materials also highlighted that promising preclinical findings do not necessarily translate into confirmed clinical benefits in humans [161]. A meta-analysis of eight randomized controlled trials showed that topical herbal products may improve skin hydration and overall elasticity while reducing melanin levels and erythema, although no significant effect was observed on TEWL. In addition, no significant improvement in skin elasticity was found in studies longer than 8 weeks. These findings should be interpreted with caution due to small sample sizes, substantial heterogeneity in the types and concentrations of plant extracts and formulations, and limited assessment of certain outcomes, including wrinkle depth and the minimal erythema dose [163]. In another study, a placebo-controlled trial was conducted in which 40 women with moderate photoaging used a 10% green tea extract cream together with oral green tea supplements for 8 weeks. As a result, an improvement in elastic tissue content was observed histologically, but no significant clinical improvement was found. Because the intervention combined topical and oral administration, the contribution of the topical formulation could not be isolated [156]. Therefore, future clinical studies should use appropriate controls, adequate sample sizes, standardized measurements, and sufficient follow-up periods to confirm the cosmetic efficacy of natural compounds.

### 3.7. Future Perspectives

Natural bioactive compounds present a promising platform for developing new cosmeceuticals in the near future. However, according to this review, as highlighted above, there are various limitations when they are used in cosmetic formulations, including variability in natural sources, lack of standardization, low stability, poor skin penetration, and the limited number of clinical studies. Among these, the limited availability of raw materials, low formulation stability and the lack of standardized clinical evidence are currently the main barriers. Therefore, future studies should focus on improving environmentally friendly green extraction techniques for obtaining bioactive compounds with the highest yield, developing efficient delivery systems, and conducting well-designed clinical studies with standardized safety and efficacy measurements. In addition, the valorization of plant waste [164], as well as the use of microorganisms [165] and insects [166], may provide sustainable sources of active metabolites. Furthermore, advanced analytical techniques, such as Ultra-Performance Liquid Chromatography–Mass Spectrometry (UPLC-MS) based metabolic profiling, high-resolution mass spectrometry and NMR spectroscopy, can facilitate the identification and characterization of secondary metabolites from diverse biological sources [167,168]. Their biological activities should then be evaluated using appropriate experimental models, supported by in silico approaches such as molecular docking and ADMET prediction, to identify compounds with potential cosmeceutical applications [167,168]. Moreover, clear regulatory guidelines are necessary to ensure the safety and effectiveness of cosmeceutical products. The use of modern skin evaluation methods, such as the Franz diffusion cell, patch testing [54,169], and 3D human skin models, can help assess skin penetration, release, efficacy, and safety of cosmeceuticals while reducing the need for animal testing.

## 4. Conclusions

Bioactive compounds are capable of regulating multiple cellular pathways that are related to oxidative stress, inflammation, skin aging, melanogenesis, and skin barrier functions at the same time. Because of their diverse biological activities, they have become one of the most significant classes of compounds for the healthcare and cosmetics sectors. Evidence shows that the effectiveness of these compounds is not determined by their biological activity, but also depends on the choice of natural source, extraction and purification methods, formulation design, novel delivery systems, product stability, and bioavailability. Any future developments within this field will require green extraction methods for plant species with the highest yield of bioactive compounds, advanced delivery systems, and well-designed clinical studies to ensure consistent quality, safety, and efficacy. Such multidisciplinary efforts will support the development of scientifically validated, high-performance, and environmentally sustainable cosmeceutical products.

## Figures and Tables

**Table 1:** Most important polysaccharides used in cosmetic and skin care products.

Polysaccharide	Source	Cosmetic Application	Reference
Pectin	Plant cell walls especially citrus fruits and apple pomace, sugar beet, and sunflower processing	Emulsifying and gelling agent, anti-aging agent, and stabilizer in shampoos and lotions	[128,129]
Starch	Corn, potato, rice, wheat, oats, sorghum, barley	Thickening agent, gelling agent, stabilizing agent, moisture retention, and skin conditioning	[130,131]
Cellulose	Plant (with hemicelluloses)	Absorbent, thickening agent, and texture enhancer; moisture retention and skin tissue repair products	[131,132]
Bacterial Cellulose	*Komagataeibacter* (formerly *Acetobacter*) spp.	Facial masks, wound dressings, implants, hydrogel,	[133]
Hyaluronic Acid	Connective tissues, bacteria	Moisturizer, anti-aging ingredient	[134]
Chitin/Chitosan	Fungi, yeasts, krill, lobsters, crabs, shrimps, and insects	Film forming, hair fixing, wound dressings, moisture retention	[121,135]
Alginate	Marine brown algae	Hydrogel, facial masks, thickener, moisturizing and protective effect on skin	[136,137]
Carrageenan	*K. alvarezii*, *K. striatum*, and *E. spinosum*	Facial masks, thickener, stabilizer, film-forming agent	[138,139]
Fucoidan	Brown algae	Anti-aging and skin care formulations	[140]
Ulvan	Green algae (*Ulva* spp.)	Stabilizing agent, moisturizing	[141,142]
Xanthan Gum	*Xanthomonas campestris*	Thickener, stabilizer, suspending agent	[131]
Pullulan	*Aureobasidium pullulan*	Thickener, colorant, preservative, skin-whitening creams, water retention, gas-barrier properties, and regenerative properties	[143]
	*Pullularia pullulans*		

## Data Availability

No new data were created or analyzed in this study. Data sharing is not applicable to this article.

## Abbreviations

ABTS	2,2′-Azino-bis(3-ethylbenzothiazoline-6-sulfonic acid)
ARE	Antioxidant Response Element
ATP	Adenosine Triphosphate
BSF	Black Soldier Fly
CAT	Catalase
cAMP	Cyclic Adenosine Monophosphate
CER[EOS]	Esterified Omega-Hydroxyacyl Sphingosine Ceramide
CGRP	Calcitonin Gene-Related Peptide
COX-2	Cyclooxygenase-2
DNA	Deoxyribonucleic Acid
DPPH	2,2-Diphenyl-1-picrylhydrazyl
EC	Epicatechin
ECG	Epicatechin Gallate
EGC	Epigallocatechin
EGCG	Epigallocatechin Gallate
GL	Glycyrrhizic Acid
GPx	Glutathione Peroxidase
GSH	Glutathione
HMGB1	High Mobility Group Box 1
HO-1	Heme Oxygenase-1
HPLC	High-Performance Liquid Chromatography
iNOS	Inducible Nitric Oxide Synthase
MAE	Microwave-Assisted Extraction
MAPK	Mitogen-Activated Protein Kinase
MDA	Malondialdehyde
MMP	Matrix Metalloproteinase
NAD+	Nicotinamide Adenine Dinucleotide
NF-κB	Nuclear Factor Kappa B
NLCs	Nanostructured Lipid Carriers
NLRP3	NOD-, LRR- and Pyrin Domain-Containing Protein 3
NO	Nitric Oxide
NOX2	NADPH Oxidase 2
Nrf2	Nuclear Factor Erythroid 2-Related Factor 2
PDE	Phosphodiesterase
PGE_2_	Prostaglandin E2
PLE	Pressurized Liquid Extraction
PPARα	Peroxisome Proliferator-Activated Receptor Alpha
PPARγ	Peroxisome Proliferator-Activated Receptor Gamma
RAR	Retinoic Acid Receptor
RNS	Reactive Nitrogen Species
ROS	Reactive Oxygen Species
RXR	Retinoid X Receptor
SFE	Supercritical Fluid Extraction
SIRT	Sirtuin
SLNs	Solid Lipid Nanoparticles
SOD	Superoxide Dismutase
STAT	Signal Transducer and Activator of Transcription
TEWL	Transepidermal Water Loss
TGF-β	Transforming Growth Factor Beta
TNF-α	Tumor Necrosis Factor Alpha
TRP	Transient Receptor Potential
TRPA1	Transient Receptor Potential Ankyrin 1
TRPM8	Transient Receptor Potential Melastatin 8
TRPV1	Transient Receptor Potential Vanilloid 1
UAE	Ultrasound-Assisted Extraction
UPLC-MS	Ultra-Performance Liquid Chromatography–Mass Spectrometry
UV	Ultraviolet
VOO	Virgin Olive Oil
